# Supplementary material for: Pharmacological inhibition of Mint3 attenuates tumour growth, metastasis, and endotoxic shock
Source: Commun Biol. 2021 Oct 7;4:1165. doi: 10.1038/s42003-021-02701-1 (PMC8497560; doi:10.1038/s42003-021-02701-1)
Supplement: Supplementary file 2 — Supplementary Information [file 42003_2021_2701_MOESM2_ESM.pdf]

Supplementary Information

Pharmacological inhibition of Mint3 attenuates tumour growth, metastasis, and endotoxic shock

Takeharu Sakamoto[1, 2]*, Yuya Fukui[2, 3], Yasumitsu Kondoh[4], Kaori Honda[4], Takeshi Shimizu[4], Toshiro Hara[5], Tetsuro Hayashi[6], Yurika Saitoh[7], Yoshinori Murakami[6], Jun-ichiro Inoue[3], Shuichi Kaneko[2], Hiroyuki Osada[4], and Motoharu Seiki[5]

[1]Department of Cancer Biology, Institute of Biomedical Science, Kansai Medical University, Shin-machi, Hirakata, Osaka, Japan.

[2]Department of System Biology, Institute of Medical, Pharmaceutical and Health Sciences, Kanazawa University, Takaramachi, Kanazawa, Ishikawa, Japan

[3]Division of Cellular and Molecular Biology, the Institute of Medical Science, The University of Tokyo, Shirokanedai, Minato-ku, Tokyo, Japan

[4]Chemical Biology Research Group, RIKEN Center for Sustainable Resource Science, Wako, Saitama, Japan

[5]Division of Cancer Cell Research, Institute of Medical Science, The University of Tokyo, Shirokanedai, Minato-ku, Tokyo, Japan

[6]Division of Molecular Pathology, Institute of Medical Science, The University of Tokyo, Shirokanedai, Minato-ku, Tokyo, Japan

[7]Center for Medical Education, Teikyo University of Science, Senjusakuragi, Adachi-ku, Tokyo, Japan

***Address correspondence to:** Takeharu Sakamoto, PhD, Department of Cancer Biology, Institute of Biomedical Science, Kansai Medical University, 2-5-1 Shin-machi, Hirakata, Osaka 573-1010, Japan. Tel: +81-72-804-2363; E-mail: sakamott@hirakata.kmu.ac.jp

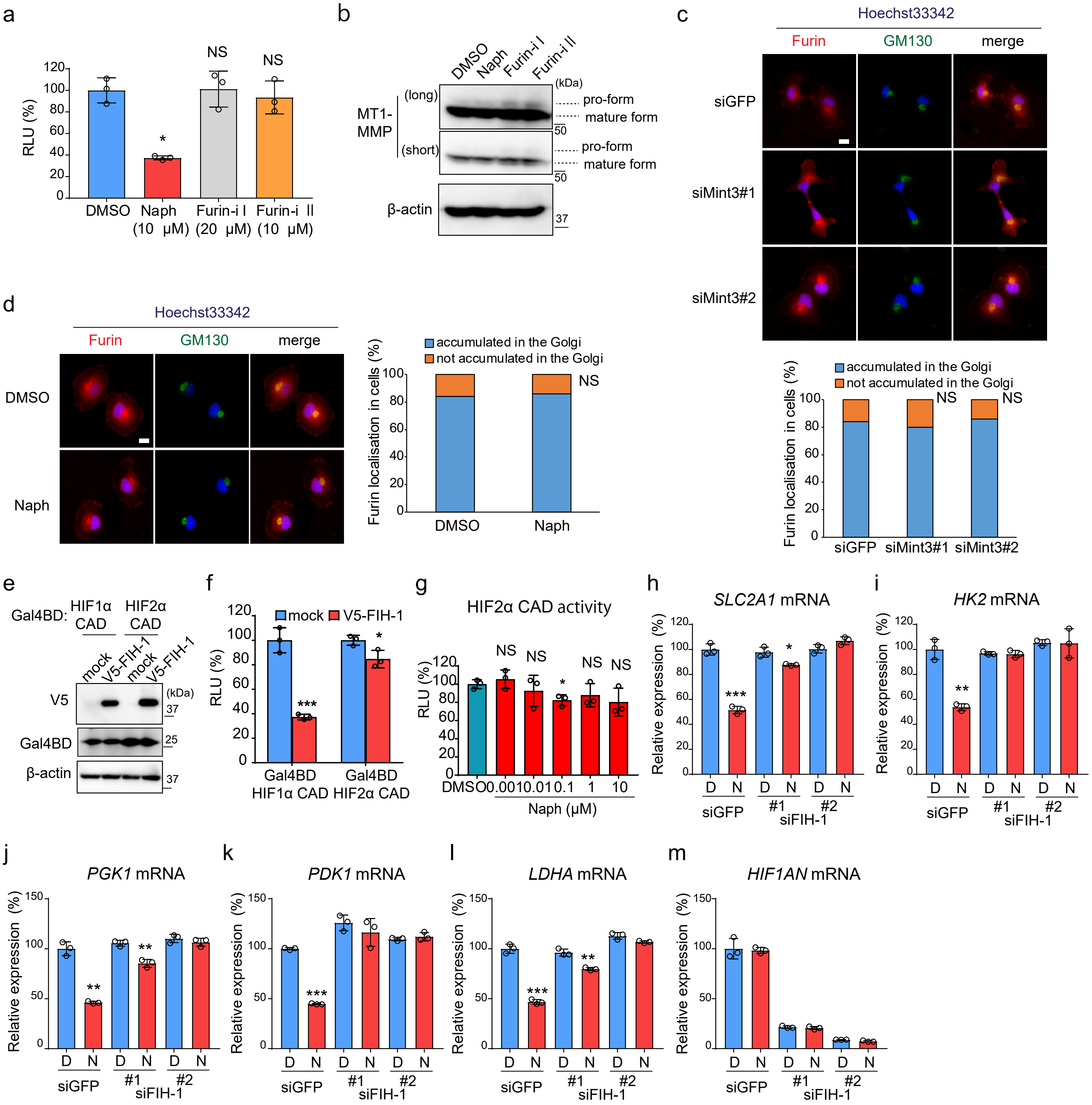

Supplementary Figure 1. Naphthofluorescein attenuates HIF-1 activity independently of furin inhibition in HT1080 cells.

(**a**) Luciferase assay for HIF-1 activity in HT1080 cells treated with DMSO, naphthofluorescein (Naph), or furin inhibitors (Furin-i, I and II) at the indicated concentrations.

(**b**) Immunoblotting of the pro-form and mature form of MT1-MMP in HT1080 cells treated with DMSO, NaphF, or Furin-i. Note that Furin-i increased pro-MT1-MMP whereas NaphF did not.

(**c**) Immunostaining of furin (red) and the Golgi marker protein GM130 (green) in HT1080 cells transfected with control siRNA (siGFP) or siRNAs against Mint3 (siMint3#1, #2). (top) Representative photos. Scale bar = 10 µm. (bottom) Cellular localisations of furin were analysed using the chi-square test with Yates' correction (n = 50). NS, not significant.

(**d**) Immunostaining of furin (red) and GM130 (green) in HT1080 treated with DMSO or Naph (10 µM) for 24 h. (left) Representative photos. Scale bar = 10 µm. (right) Cellular localisations of furin were analysed using the chi-square test with Yates' correction (n = 50). NS, not significant.

(**e**) Expression of V5-tagged FIH-1 (V5-FIH-1) and Gal4BD-fused HIF-1α CAD or HIF-2α CAD in HT1080 cells.

(**f**) Luciferase assay for HIF-1 and HIF-2 activities in siRNA-transfected HT1080 cells expressing mock or V5-FIH-1.

(**g**) Luciferase assay for HIF-2 activity in HT1080 cells treated with Naph at the indicated concentrations.

(**h**–**m**) mRNA levels of glycolysis-related HIF-1 target genes (h–l) and HIF1AN which encodes FIH-1 (m) in control (siGFP) or FIH-1-depleted (siFIH-1#1, #2) HT1080 cells treated with DMSO or Naph (10 µM) for 24 h.

In **a** and **f**–**m**, error bars indicate the SD (n = 3). Data were analysed using Student' s t-test. *p < 0.05, **p < 0.01, ***p < 0.001. NS, not significant.

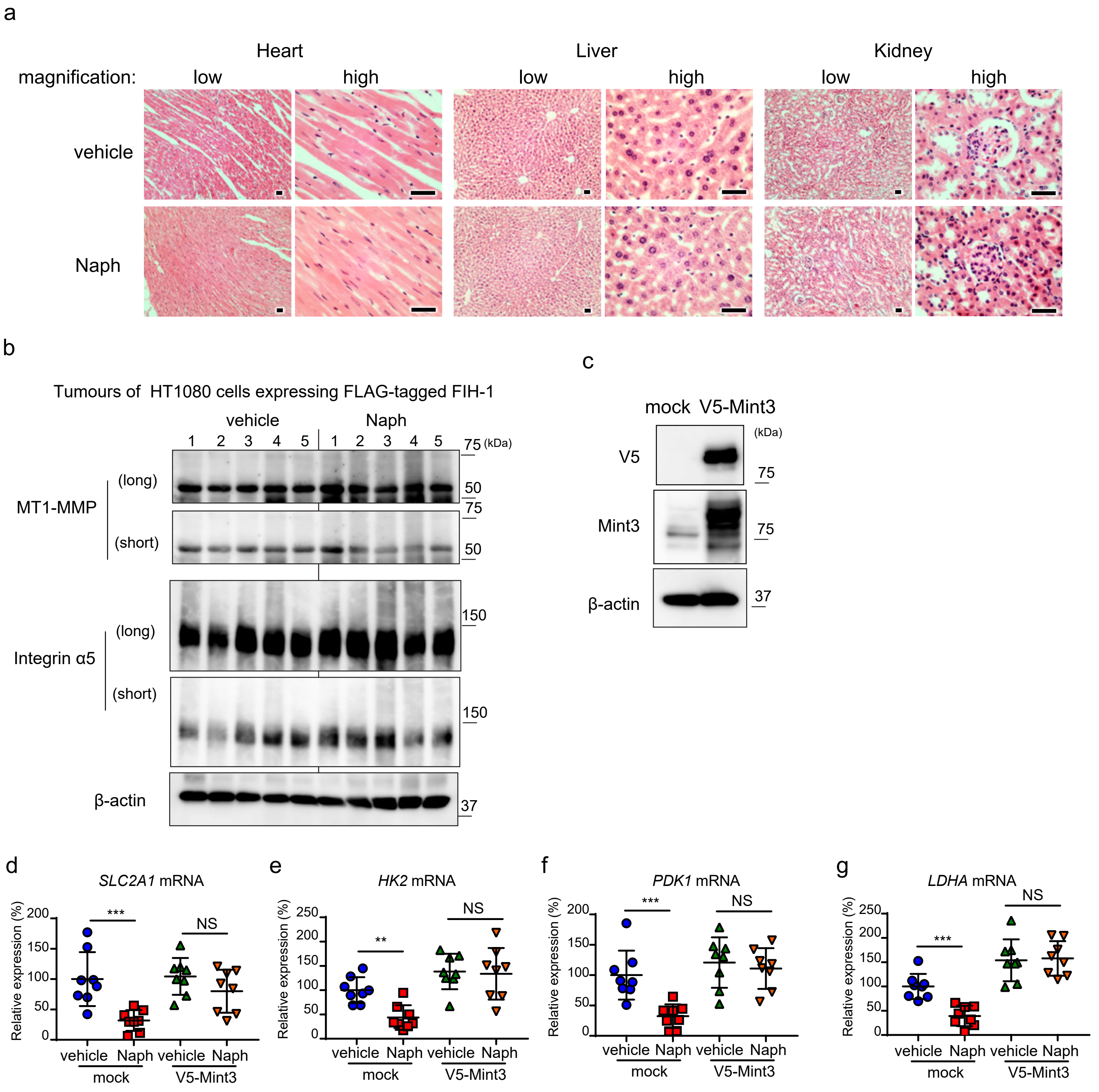

Supplementary Figure 2. Naphthofluorescein administration decreases Mint3-mediated HIF-1 target gene expression in tumours without apparent tissue damage in the lung, liver, and kidney.

(**a**) Representative images of hematoxylin and eosin-stained lung, liver, and kidney sections from vehicle - or naphthofluorescein (Naph)-injected mice that were treated for 2 weeks, as illustrated in Figure 4a. Scale bars = 50 μm.

(**b**) Immunoblotting of MT1-MMP and integrin α5 in tumours of HT1080 cells expressing FLAG-tagged FIH-1. Note that the bands of MT1-MMP and integrin α5 indicate the mature forms of MT1-MMP and integrin α5 and that Naph treatment does not increase the pro-form of these proteins in tumours.

(**c**) Expression of endogenous Mint3 and V5-tagged Mint3 (V5-Mint3) expression in HT1080 cells stably expressing mock or V5-Mint3.

(**d**–**g**) Expression of HIF-1 target genes in tumours of HT1080 cells expressing mock or V5-Mint3 6 h after Naph injection. Error bars indicate the SD (n = 8). Data were analysed using the Mann–Whitney U test. $^{**}p < 0.01$, $^{***}p < 0.001$. NS, not significant.

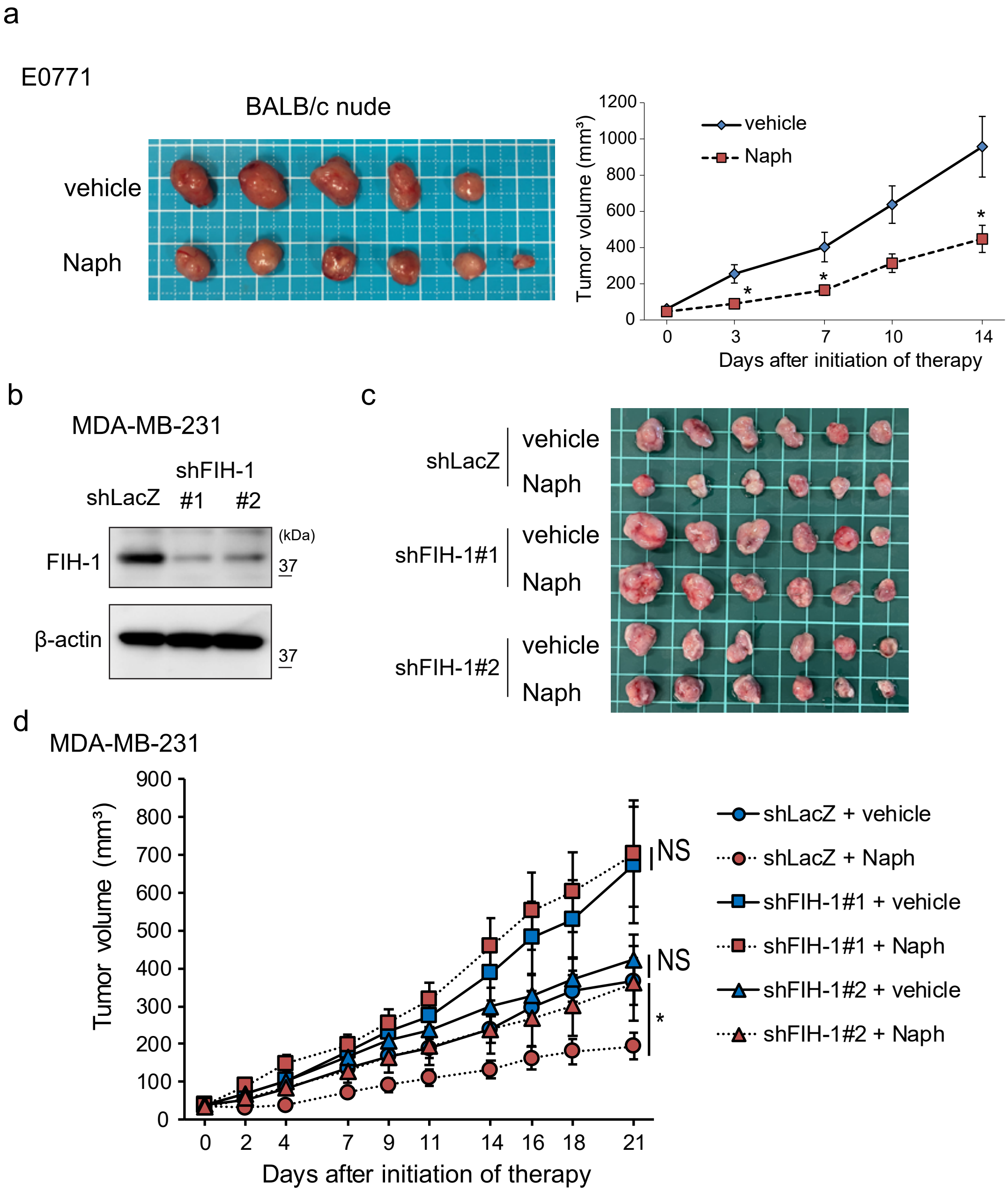

Supplementary Figure 3. Naphthofluorescein attenuates tumour growth of MDA-MB-231 cells in immunodeficient mice in an FIH-1-dependent manner. (**a**) BALB/c nude mice bearing E0771 tumours were intraperitoneally injected with vehicle or Naph (100 mg/kg b.w.) 5 times a week. (Left) Photos of tumours at Day 14 after the initiation of Naph administration. (Right) Tumour volumes of E0771 cells in mice. Data are presented as the mean ± SEM (n = 5–6). Data were analysed using the Mann–Whitney U test. *$p < 0.05$.

(**b**) Immunoblotting of FIH-1 in control (shLacZ) and FIH-1-depleted (shFIH-1#1, #2) MDA-MB-231 cells.

(**c**, **d**) Tumour growth of control (shLacZ) and FIH-1-depleted (shFIH-1#1, #2) MDA-MB-231 cells in immunodeficient mice treated with vehicle or Naph (100 mg/kg b.w.). Mice were intraperitoneally injected with vehicle or Naph, 5 times a week. (**c**) Photos of tumours at Day 21 after the initiation of Naph administration. (**d**) Tumour volumes of MDA-MB-231 cells in mice. Data are presented as the mean ± SEM (n = 6). Data were analysed using the Mann–Whitney U test. *$p < 0.05$. NS, not significant.

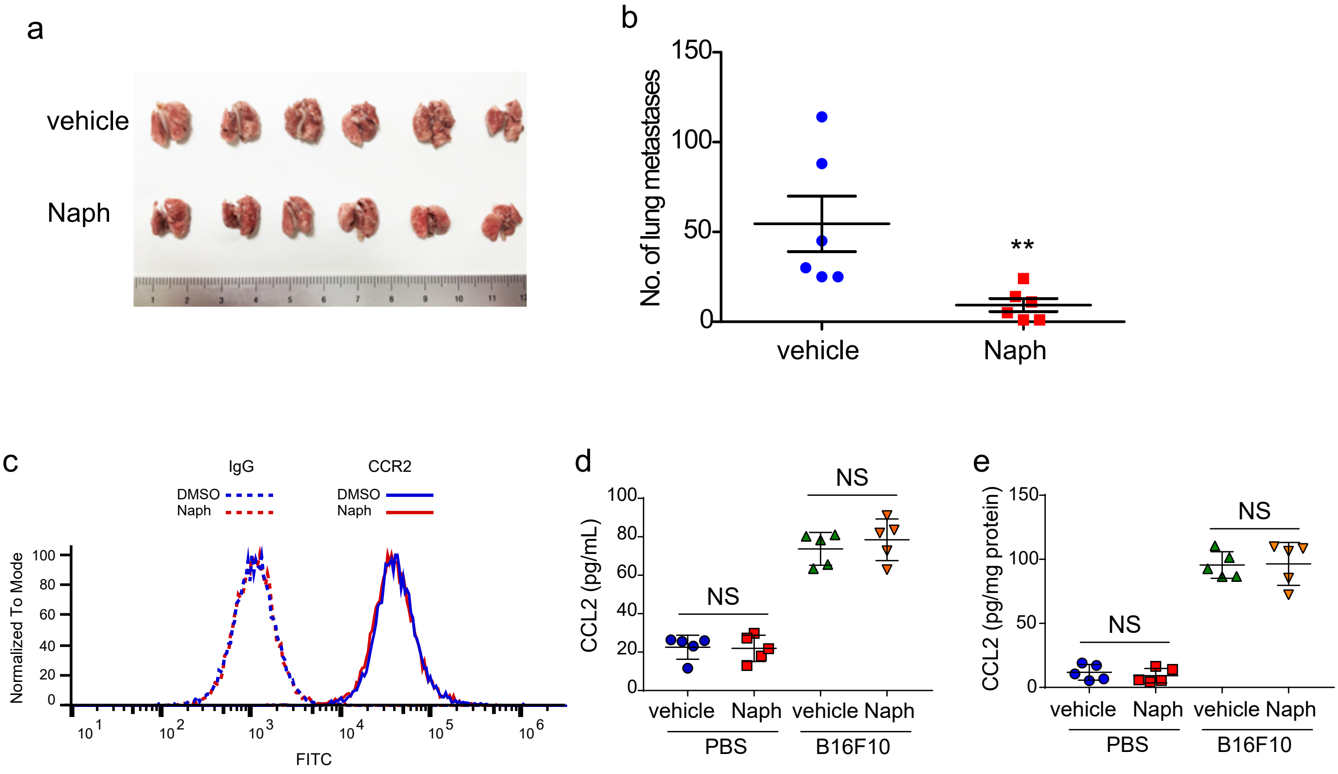

Supplementary Figure 4. Naphthofluorescein administration attenuates lung metastasis without affecting the expression of CCL2 and CCR2.
(**a**) Photos of metastatic lungs from vehicle- or NaphF-treated (100 mg/kg b.w.) mice intravenously injected with LLC cells.
(**b**) Number of metastatic foci of LLC cells in the lungs from vehicle- or NaphF-treated mice (n = 6 mice per group). Data are presented as the mean ± SEM. Data were analysed using the Mann–Whitney U test. **p < 0.01.
(**c**) Flow cytometric analysis of CCR2 expression on bone marrow-derived macrophages treated with DMSO or Naph (10 µM) for 24 h.
(**d**, **e**) Quantitative analysis of CCL2 in the peripheral blood (d) or the lungs (e) of DMSO- or Naph-treated mice at 4 h after i.v. injection with B16F10 cells or phosphate-buffered saline (PBS) (n = 5 per group). Data are presented as the mean ± SD. Data were analysed using the Mann–Whitney U test. NS, not significant.

Fig. 2a

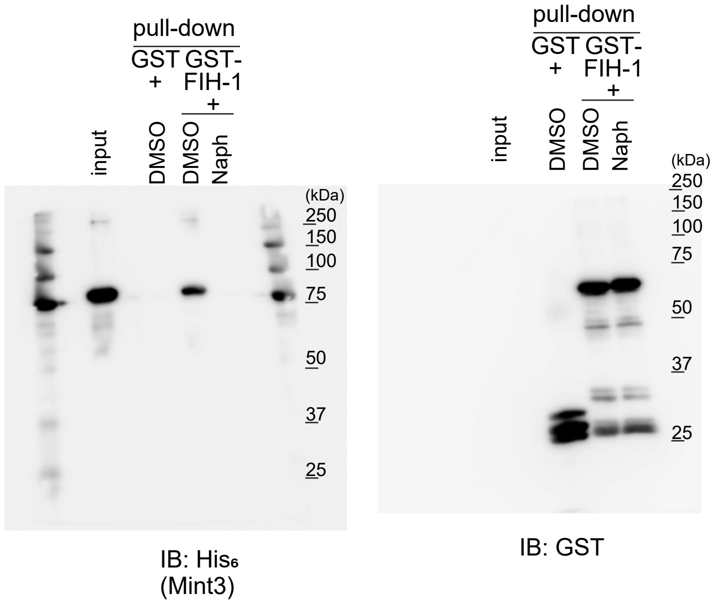

Fig. 2b

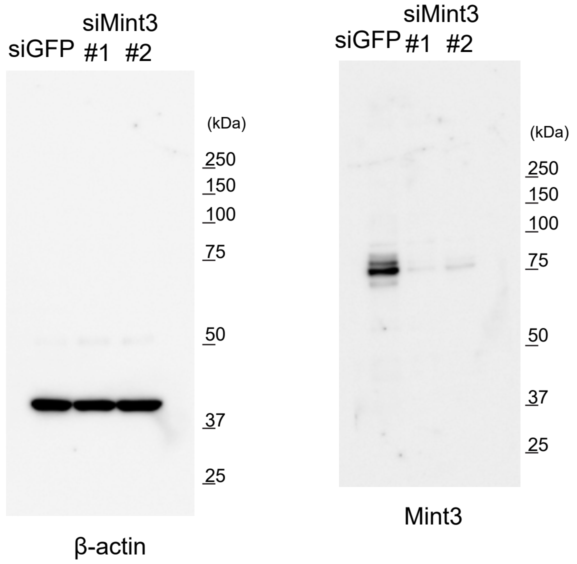

Fig. 2d

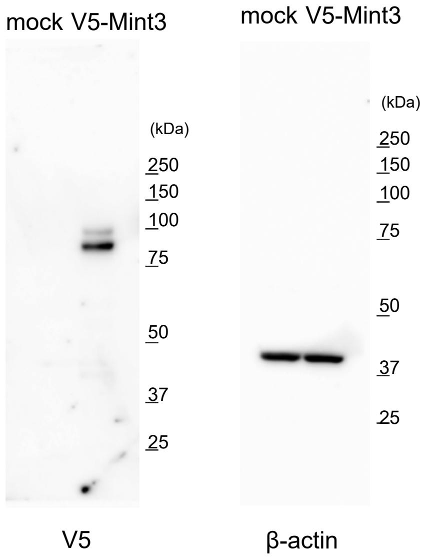

Fig. 2f

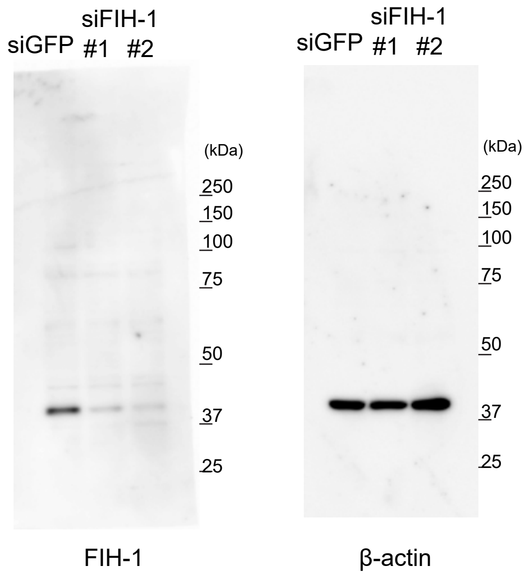

Fig. 3a

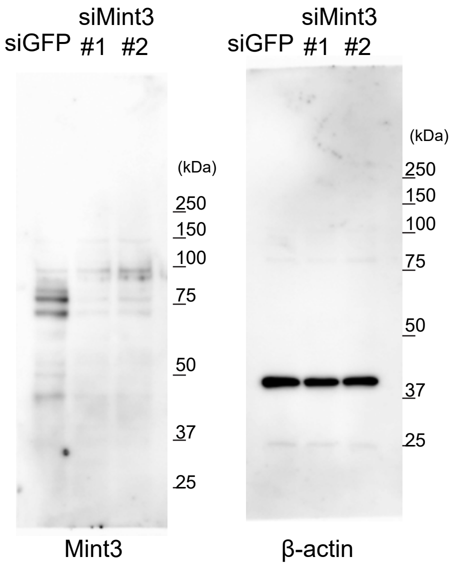

Fig. 3c

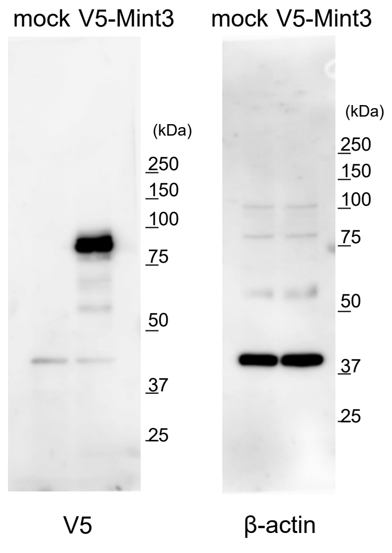

Fig. 3e

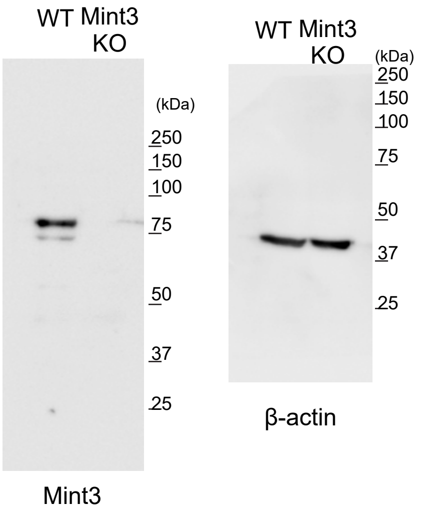

Fig. 4b

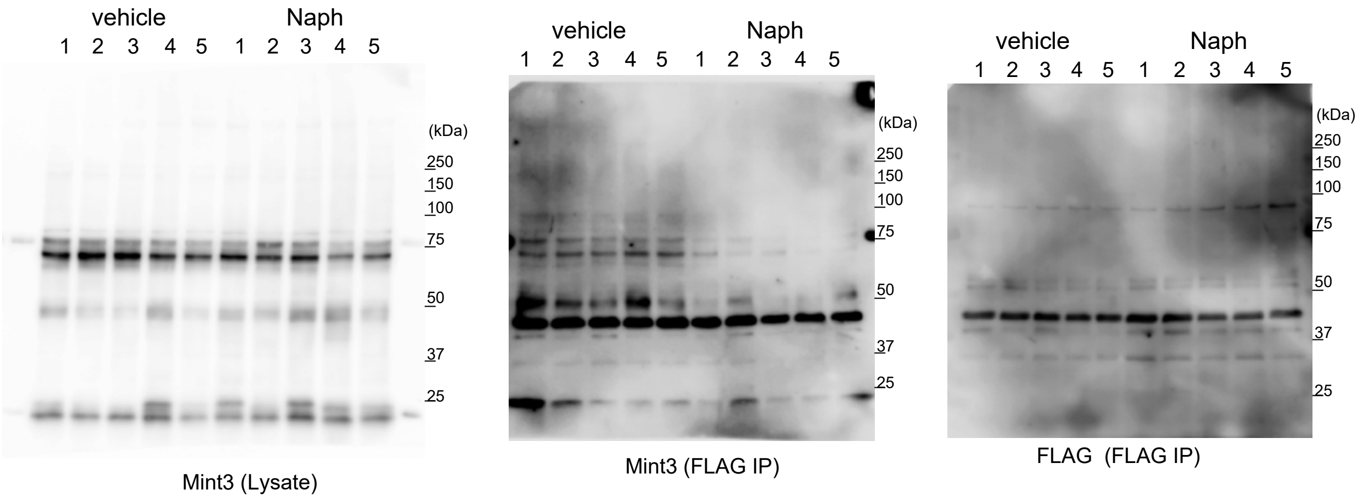

Supplementary Fig. 1b

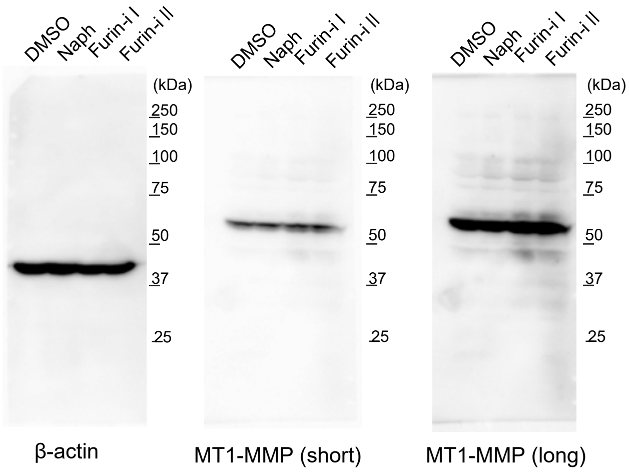

Supplementary Fig. 1e

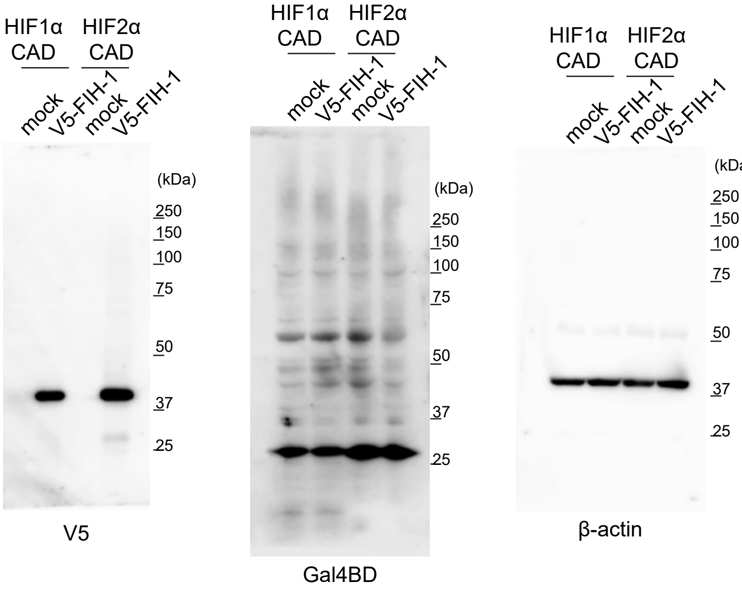

Supplementary Fig. 2b

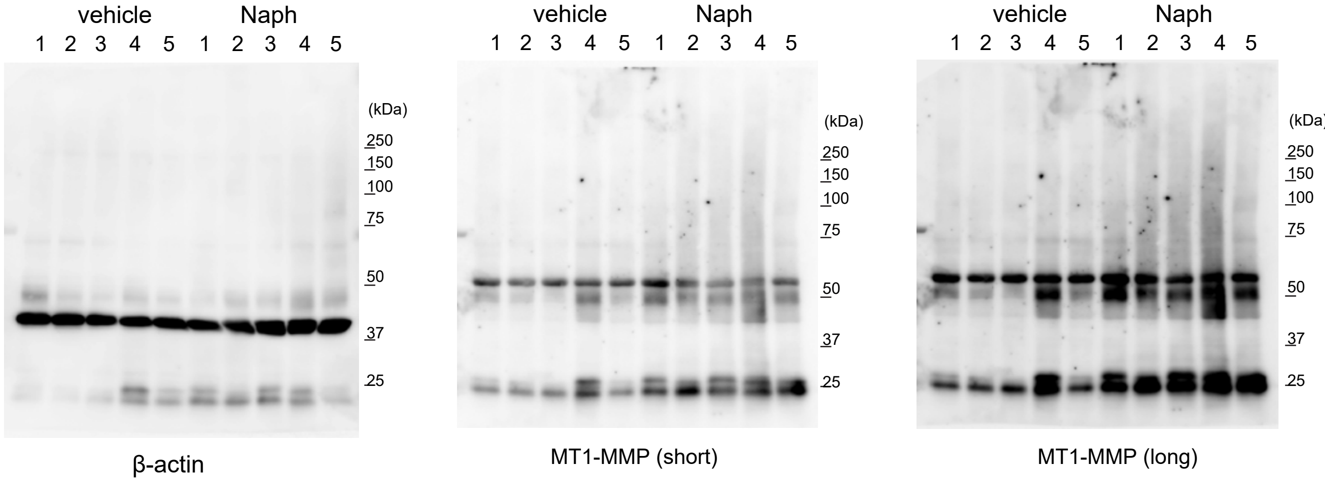

Supplementary Fig. 2b

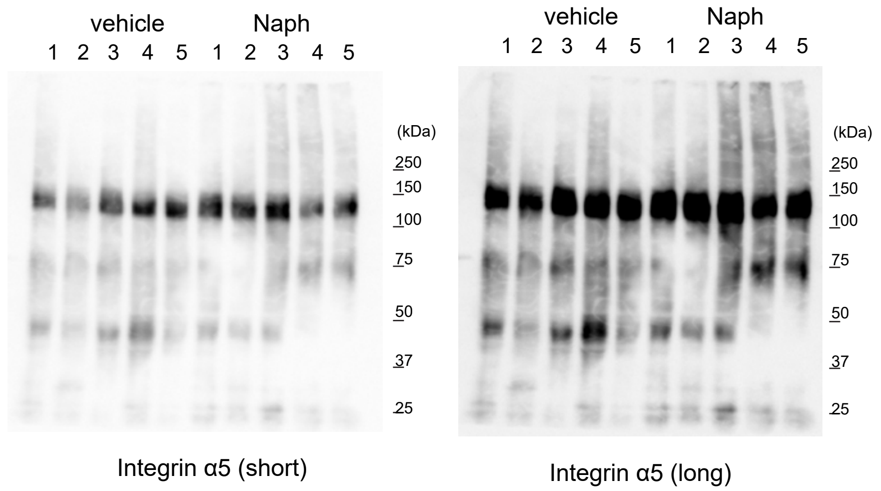

Supplementary Fig. 2c

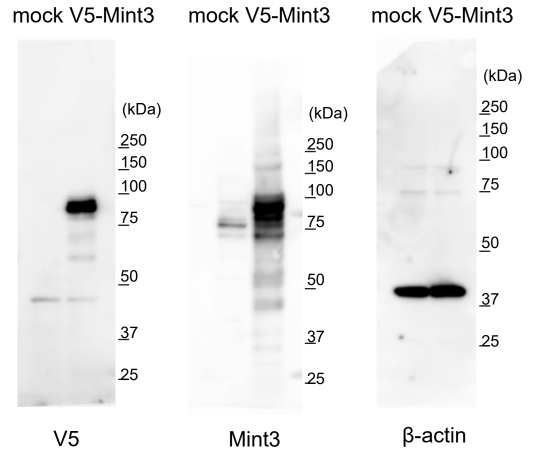

Supplementary Fig. 3b

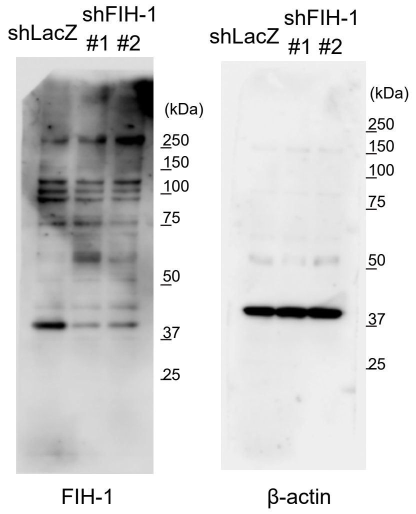

Supplementary Figure 5. Uncropped and unedited blot images.

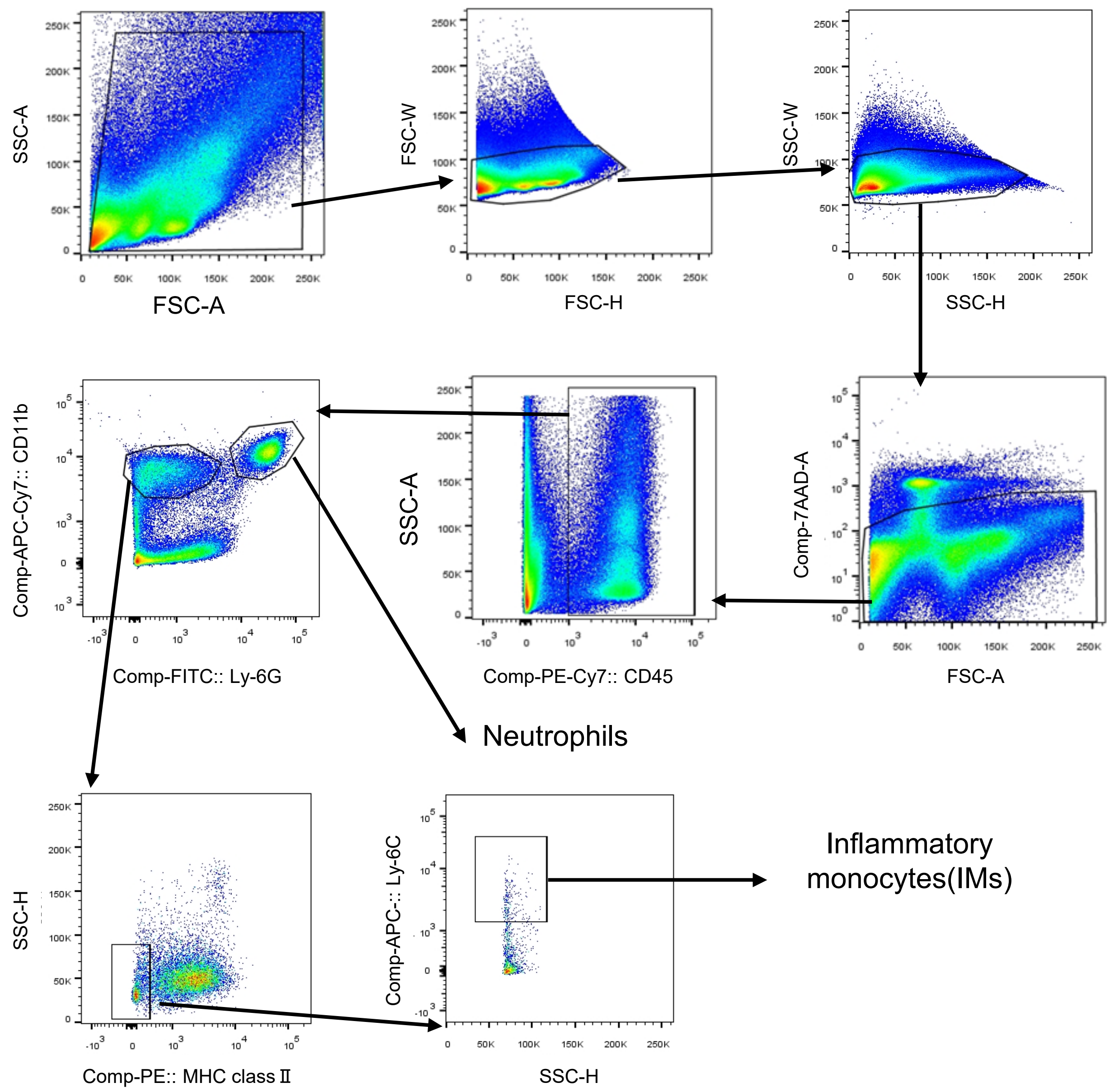

Supplementary Figure 6. Flow cytometric analysis strategy for the definition of inflammatory monocytes.

No.	Name	Structure	MW
1	NPD7801	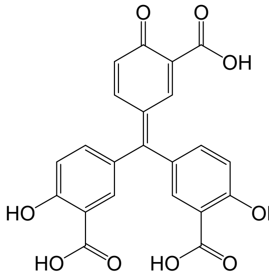	422.35
2	Chromeazurol B	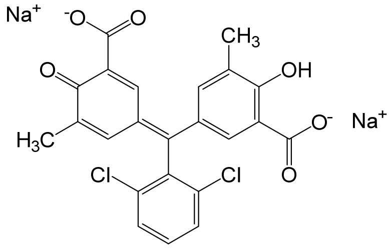	503.25
3	Chromeazurol S	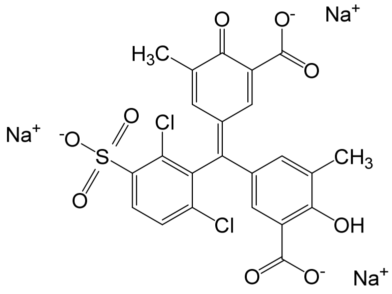	605.29
4	NPD5551	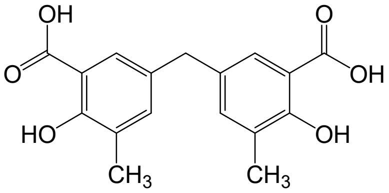	316.31
5	NPD10450	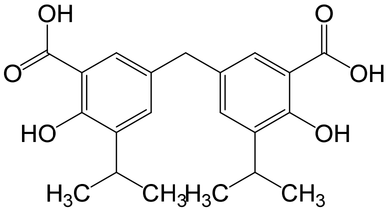	372.42
6	NPD729	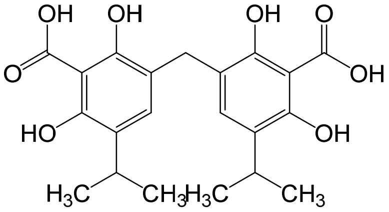	372.42

7	NPD8633	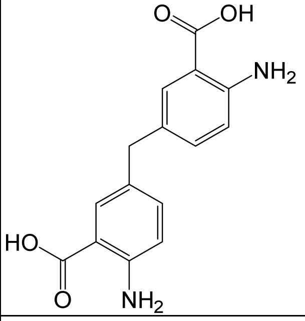	286.29
8	Pyrocatechol violet	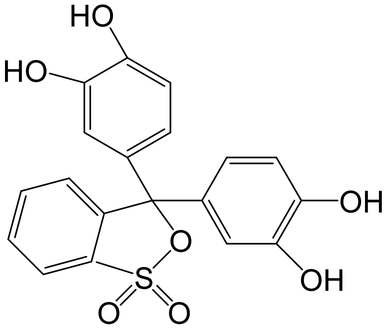	386.38
9	Gentian violet	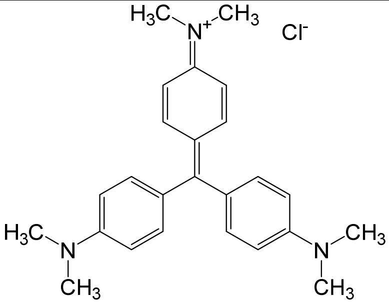	407.99
10	Fast green FCF	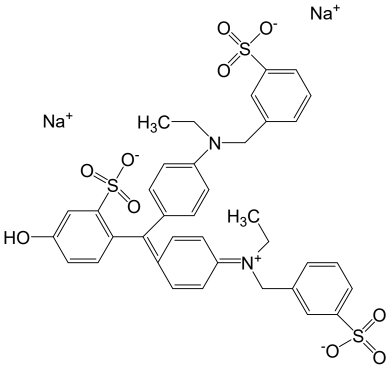	808.86
11	Bromocresol purple	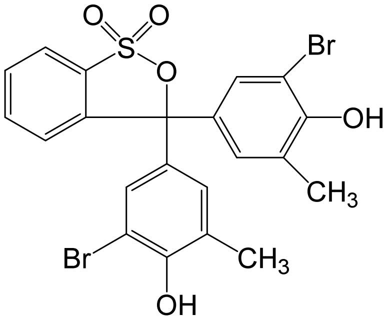	540.23

12	Bromophenol blue	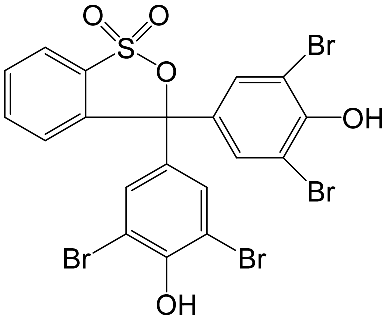	669.97
13	Phenolphtalin	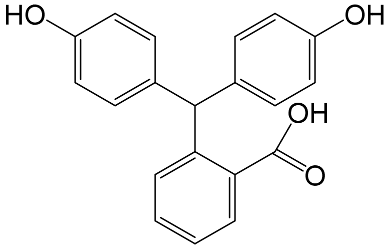	320.35
14	Phenolphtalein	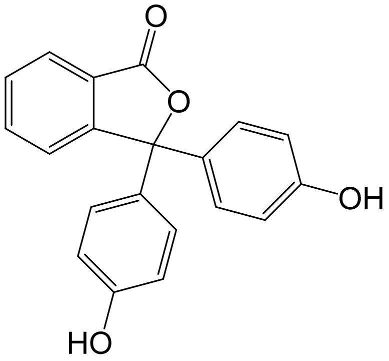	318.33
15	NPD8350	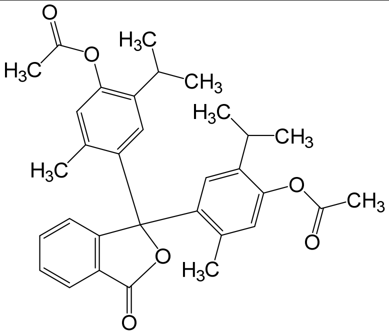	514.62
16	NPD6791	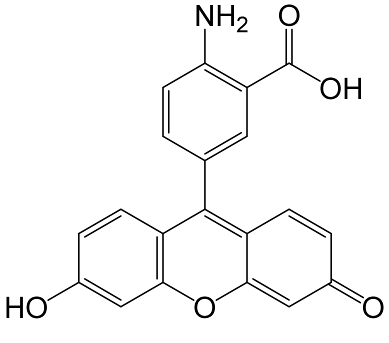	347.33

17	Fluoresceinamine isomer I	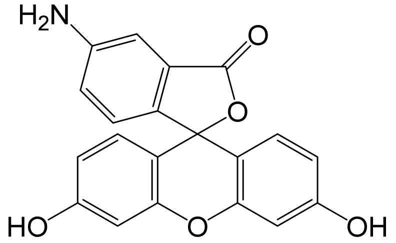	347.33
18	3-O-methylfluorescein phosphate cyclohexylammonium salt	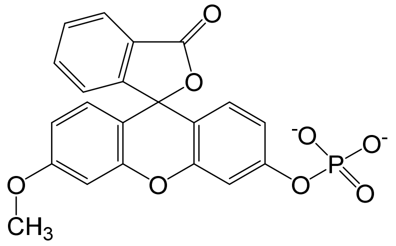	424.31
19	NPD8369 Naphthofluorescein	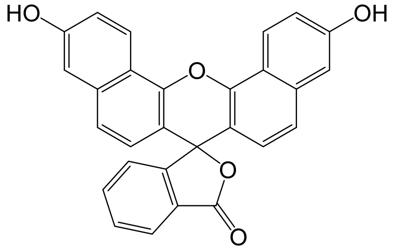	432.44

Supplementary Table 1. List of compounds tested for the HIF-1 reporter.

	vehicle (av±SD)	Naph (av±SD)	p value
TP (g/dL)	6.01±0.14	5.70±0.28	0.0649
ALB (g/dL)	3.80±0.06	3.62±0.21	0.1126
BUN (mg/dL)	36.08±1.91	28.01±3.69	0.2251
CRE (mg/dL)	0.158±0.021	0.151±0.013	0.5498
Na (mEq/L)	158.5±1.3	158.5±1.2	>0.999
K (mEq/L)	7.3±0.6	7.4±0.8	0.8485
Cl (mEq/L)	116.66±1.96	116.83±1.16	>0.999
Ca (mg/dL)	9.86±0.16	9.95±0.13	0.5238
IP (mg/dL)	10.03±0.71	10.58±1.14	0.4848
AST (IU/L)	185.83±36.35	153.00±27.28	0.1212
ALT (IU/L)	33.17±5.19	29.83±4.02	0.2241
LDH (IU/L)	668.00±92.31	786.66±173.21	0.3939
AMY (IU/L)	2661.16±241.73	2418.33±224.16	0.1797
r-GT (IU/L)	3 >	3 >	ND
T-CHO (mg/dL)	65.00±1.78	67.33±4.76	0.3290
TG (mg/dL)	55.67±17.96	39.16±11.03	0.0606
HDL-C (mg/dL)	41.16±1.83	41.33±2.50	0.9784
T-BIL (mg/dL)	0.051±0.009	0.048±0.017	0.8377
GLU (mg/dL)	132.00±10.55	115.16±20.39	0.0844

Supplementary Table 2. Biochemical analysis of sera from vehicle - or Naph-treated mice. Data are presented as mean ± SD (n = 6 per group) and were analysed by the Mann–Whitney U-test. ND, not determined.
