# Supplementary material for: Pharmacological inhibition of Mint3 attenuates tumour growth, metastasis, and endotoxic shock
Source: Commun Biol. 2021 Oct 7;4:1165. doi: 10.1038/s42003-021-02701-1 (PMC8497560; doi:10.1038/s42003-021-02701-1)
Supplement: Supplementary file 3 — Description of Additional Supplementary Files [file 42003_2021_2701_MOESM3_ESM.pdf]

Description of Additional Supplementary Files

File name: Supplementary Data 1

Description: Data source file.
