# Supplementary material for: Pharmacological inhibition of Mint3 attenuates tumour growth, metastasis, and endotoxic shock
Source: Commun Biol. 2021 Oct 7;4:1165. doi: 10.1038/s42003-021-02701-1 (PMC8497560; doi:10.1038/s42003-021-02701-1)
Supplement: Supplementary file 5 — Reporting Summary [file 42003_2021_2701_MOESM5_ESM.pdf]

COMMSBIO-21-0269B
Corresponding author(s): Takeharu Sakamoto
Last updated by author(s): 2021/09/01

Reporting Summary

Nature Research wishes to improve the reproducibility of the work that we publish. This form provides structure for consistency and transparency in reporting. For further information on Nature Research policies, see our Editorial Policies and the Editorial Policy Checklist.

Statistics

For all statistical analyses, confirm that the following items are present in the figure legend, table legend, main text, or Methods section.

n/a	Confirmed	
☐	☒	The exact sample size (n) for each experimental group/condition, given as a discrete number and unit of measurement
☐	☒	A statement on whether measurements were taken from distinct samples or whether the same sample was measured repeatedly
☐	☒	The statistical test(s) used AND whether they are one- or two-sided<br>*Only common tests should be described solely by name; describe more complex techniques in the Methods section.*
☒	☐	A description of all covariates tested
☒	☐	A description of any assumptions or corrections, such as tests of normality and adjustment for multiple comparisons
☐	☒	A full description of the statistical parameters including central tendency (e.g. means) or other basic estimates (e.g. regression coefficient) AND variation (e.g. standard deviation) or associated estimates of uncertainty (e.g. confidence intervals)
☐	☒	For null hypothesis testing, the test statistic (e.g. F, t, r) with confidence intervals, effect sizes, degrees of freedom and P value noted<br>*Give P values as exact values whenever suitable.*
☒	☐	For Bayesian analysis, information on the choice of priors and Markov chain Monte Carlo settings
☒	☐	For hierarchical and complex designs, identification of the appropriate level for tests and full reporting of outcomes
☒	☐	Estimates of effect sizes (e.g. Cohen's d, Pearson's r), indicating how they were calculated

Our web collection on statistics for biologists contains articles on many of the points above.

Software and code

Policy information about availability of computer code

Data collection	BD FACSuite software; Olympus FLUOVIEW; GE Healthcare ImageQuant LAS4000; Applied Biosystems SDS Software
Data analysis	GraphPad Prism (ver. 7);TreeStar FlowJo (ver. 10); the R package “survival” and “survminer”

For manuscripts utilizing custom algorithms or software that are central to the research but not yet described in published literature, software must be made available to editors and reviewers. We strongly encourage code deposition in a community repository (e.g. GitHub). See the Nature Research guidelines for submitting code & software for further information.

Data

Policy information about availability of data

All manuscripts must include a data availability statement. This statement should provide the following information, where applicable:
- Accession codes, unique identifiers, or web links for publicly available datasets
- A list of figures that have associated raw data
- A description of any restrictions on data availability

Figure Source File is available in the Supplementary Information. There is no restriction on data availability.

Field-specific reporting

Please select the one below that is the best fit for your research. If you are not sure, read the appropriate sections before making your selection.

☒ Life sciences ☐ Behavioural & social sciences ☐ Ecological, evolutionary & environmental sciences

For a reference copy of the document with all sections, see nature.com/documents/nr-reporting-summary-flat.pdf

Life sciences study design

All studies must disclose on these points even when the disclosure is negative.

Sample size	Sample size is based on statistical analysis of variance and on exploratory experiments.
Data exclusions	No data was excluded.
Replication	Each in vitro experiment was replicated at least three times successfully. Animal experiments were replicated at least twice successfully.
Randomization	For animal experiments, mice were randomly assigned to each experimental group.
Blinding	Investigators were not blinded to mouse genotypes for planning of experiments. Tumour volume of each group was blindly measured by the lab members who were not responsible for the project.

Reporting for specific materials, systems and methods

We require information from authors about some types of materials, experimental systems and methods used in many studies. Here, indicate whether each material, system or method listed is relevant to your study. If you are not sure if a list item applies to your research, read the appropriate section before selecting a response.

Materials & experimental systems

n/a	Involved in the study	
☐	☒	Antibodies
☐	☒	Eukaryotic cell lines
☒	☐	Palaeontology and archaeology
☐	☒	Animals and other organisms
☒	☐	Human research participants
☒	☐	Clinical data
☒	☐	Dual use research of concern

Methods

n/a	Involved in the study	
☒	☐	ChIP-seq
☐	☒	Flow cytometry
☒	☐	MRI-based neuroimaging

Antibodies

Antibodies used

anti-β-actin, mouse, FUJIFILM Wako, 0111-24554, 1:5000 (WB)
anti-Mint3, mouse, BD Biosciences, 611380, 1:1000 (WB)
anti-FIH-1, goat, SantaCruz, SC-26219, 1:250 (WB)
anti-His6, mouse, Roche, 11922416001, 1:1000 (WB)
anti-GST, mouse, GeneTex, GTX70195 1:1000 (WB)
anti-FLAG rabbit, Millipore, F7425, 1:1000 (WB)
anti-MT1-MMP, rabbit, Millipore, AB6004, 1:1000 (WB)
anti-Integrin alpha 5, mouse, BD Biosciences, 51-9001996, 1:1000 (WB)
anti-CD8α, rabbit, CST, 98941, 1:200 (IHC)
anti-Furin, rabbit, Abcam, ab3467, 1:200 (IFA)
anti-GM130, mouse, BD Biosciences, 610822, 1:200 (IFA)
anti-E-selectin (CD62E), rat, Abcam, ab2497, 1:200 (IFA)
anti-cleaved caspase-3, rabbit, CST, 9665, 1:100 (IFA)
anti-rabbit IgG, HRP linked whole Ab, donkey, GE Helthcare, NA934V, 1:3000 (WB)
anti-mouse IgG, HRP- linked whole Ab, sheep, GE Helthcare, NA931V, 1:3000 (WB)
Peroxidase-conjugated anti-Goat IgG(H+L), rabbit, Proteintech, SA00001-4, 1:5000 (WB)
anti-mouse IgG, Alexa Fluor 488, goat, Thermo Fischer Scientific, A-11029, 1:2000 (IFA)
anti-rabbit IgG, Alexa Fluor 546, goat, Thermo Fischer Scientific, A-11035, 1:2000 (IFA)
anti-rabbit IgG, Alexa Fluor 488, goat, Thermo Fischer Scientific, A-11034, 1:500 (IFA)
anti-rat IgG, Alexa Fluor 594, goat, Thermo Fischer Scientific, A-11007, 1:500 (IFA)
anti-mouse Ly-6G, FITC conjugate, rat, TONBO biosciences, 35-1276 1:400 (FACS)
anti-mouse MHC Class II(I-A/I-E), PE conjugate, rat, TONBO biosciences, 50-5321, 1:200 (FACS)
anti-mouse Ly-6C, APC conjugate, rat, eBioscience, 17-5932, 1:200 (FACS)
anti-mouse/human CD11b, APC-Cy7 conjugate, rat, BioLegend, 101226, 1:200 (FACS)

	anti-mouse CD45, PE-Cy7 conjugate, rat, eBioscience, 25-0451-82, 1:2000 (FACS)<br>anti-mouse CCR2, FITC conjugate, rat, BioLegend, 150607, 1:200 (FACS)<br>IgG2b, κ Isotype, FITC conjugate, rat, BioLegend, 400605, 1:200 (FACS)
Validation	All antibodies were sold by the manufacturer with validation data and citations, and they detected the specified targets in our study as expected.

Eukaryotic cell lines

Policy information about cell lines

Cell line source(s)	B16F10 and LLC cells were obtained from the Cell Resource Center for Biomedical Research, Institute of Development, Aging and Cancer, Tohoku University (Miyagi, Japan).<br>HT1080, MDA-MB-231, and AsPC-1 cells were obtained from the American Type Culture Collection (Manassas, VA, USA).
Authentication	None of the cell lines used are authentificated
Mycoplasma contamination	All cell lines were negative for Mycoplasma contamination.
Commonly misidentified lines (See ICLAC register)	No commonly misidentified line was used in this study.

Animals and other organisms

Policy information about studies involving animals; ARRIVE guidelines recommended for reporting animal research

Laboratory animals	Eight-week-old male C57BL/6J mice for the toxicity test. Six-week-old female BALB/c nude mice and C57BL/6J mice for tumor implantation analyses. 6-week-old male C57BL/6J mice for lung metastasis assays. Eight-week-old male C57BL/6J (wild-type) and Mint3 KO mice for the LPS-indued endotoxic shock assay.
Wild animals	NA
Field-collected samples	NA
Ethics oversight	the Animal Care and Use Committees of the Institute of Medical Science, University of Tokyo (PA18-18) and Institute of Medical, Pharmaceutical and Health Sciences, Kanazawa University (AP-204171).

Note that full information on the approval of the study protocol must also be provided in the manuscript.

Flow Cytometry

Plots

Confirm that:

☒ The axis labels state the marker and fluorochrome used (e.g. CD4-FITC).

☒ The axis scales are clearly visible. Include numbers along axes only for bottom left plot of group (a 'group' is an analysis of identical markers).

☒ All plots are contour plots with outliers or pseudocolor plots.

☒ A numerical value for number of cells or percentage (with statistics) is provided.

Methodology

Sample preparation	Cells from mouse lungs were isolated by manually disruption in buffer as described in Methods.
Instrument	BD Bioscience FACSVerse
Software	BD FACSuite software and TreeStar FlowJo
Cell population abundance	NA
Gating strategy	Detailed gating strategy is described in the Supplementary Information.

☒ Tick this box to confirm that a figure exemplifying the gating strategy is provided in the Supplementary Information.
